# Cardiovascular co-morbidity in patients with rheumatoid arthritis: a narrative review of risk factors, cardiovascular risk assessment and treatment

**DOI:** 10.1186/s41927-018-0014-y

**Published:** 2018-04-11

**Authors:** Aprajita Jagpal, Iris Navarro-Millán

**Affiliations:** 1 Division of Clinical Immunology and Rheumatology, University of Alabama at Birmingham, 836 Faculty Office Tower, 510 20th Street South, Birmingham, AL 35294 USA; 2 Joan and Sanford I Weill Medical College of Cornell University, Division of General Internal Medicine, 525 East 68th Street, F-2019, PO Box #331, New York, NY 10065 USA; 3 Division of Rheumatology, Hospital for Special Surgery, New York, NY USA

**Keywords:** Cardiovascular disease, Rheumatoid arthritis, Mortality, Myocardial infarction, Cardiovascular risk assessment

## Abstract

Cardiovascular disease (CVD) is markedly increased in patients with rheumatoid arthritis partly due to accelerated atherosclerosis from chronic inflammation. Traditional cardiovascular risk factors such as hypertension, hyperlipidemia, smoking, diabetes mellitus and physical inactivity are also highly prevalent among patients with rheumatoid arthritis (RA) and contribute to the CVD risk. The impact of traditional risk factors on the CVD risk appears to be different in the RA and non-RA population. However, hyperlipidemia, diabetes mellitus, body mass index and family history of CVD influence the CVD risk in RA patients the same way they do for the non-RA population. Despite that, screening and treatment of these risk factors is suboptimal among patients with RA. Recent guidelines from the European League Against Rheumatism (EULAR) recommend aggressive management of traditional risk factors in addition to RA disease activity control to decrease the CVD risk. Several CVD risk calculators are available for clinical use to stratify a patients’ risk of developing a CVD event. Most of these calculators do not account for RA as a risk factor; thus, a multiplication factor of 1.5 is recommended to predict the risk more accurately. In order to reduce CVD in the RA population, national guidelines for the prevention of CVD should be applied to manage traditional risk factors in addition to aggressive control of RA disease activity. While current data suggests a protective effect of non-biologic disease modifying anti-rheumatic drugs (DMARDs) and biologics on cardiovascular events among patients with RA, more data is needed to define this effect more accurately.

## Background

Rheumatoid arthritis (RA) is a chronic systemic inflammatory condition which leads to joint damage and physical disability [1]. Compared to the general population, a considerably higher risk of cardiovascular disease (CVD) is seen in patients with RA [2–4]. Hyperlipidemia, diabetes mellitus, family history of CVD, and body mass index are the risk factors associated with CVD risk in these patients [5]. Previous studies indicated that these traditional CVD risk factors do not fully explain the increased CVD risk among RA patients [6]. For example, a prospective cohort study of 114,342 women participating in the Nurses’ Health Study found > 2-fold higher risk of myocardial infarction in women with RA compared to non-RA, even after adjusting for cardiovascular risk factors [7]. This data suggests that RA related factors, possibly inflammation, are also associated with the increased CVD risk that exists in this population [8–12]. Thus, adequate control of RA disease activity as well as management of CVD risk factors are needed to mitigate the heightened CVD risk in RA. This is reflected in the recently published treatment guidelines from the European League Against Rheumatism (EULAR), which emphasize the importance of management of traditional CVD risk factors alongside the RA management [13].

In order to implement preventive measures, CVD risk stratification is the initial step to determine a patients’ overall risk for a CVD event. There are several CVD risk prediction models that are used for this purpose. These models were developed in the non-RA population and their accuracy stratifying CVD risk for patients with RA is still a matter of research. Attempts to develop and validate CVD risk prediction models that stratify CVD risk more accurately in patients with RA are on-going [14, 15]. This narrative review summarizes current data about CVD risk in patients with RA, the status of current CVD risk prediction models, and discusses management to reduce this risk. As such, this narrative review does not address risk of bias of the articles included and it may not have taken into consideration all the available data, as a systematic review would have done.

### Mortality/morbidity from cardiovascular disease in RA

Rheumatoid arthritis patients suffer from excess mortality from cardiovascular disease [7, 16]. CVD is the leading cause of death even in the general population; however, RA is associated with an increased risk of developing CVD by almost two fold, a risk magnitude comparable to that of diabetes mellitus [17, 18]. RA patients are twice as likely to experience a silent myocardial infarction compared to non-RA subjects [4] and carry a higher burden of coronary plaques even in the absence of clinical history of coronary artery disease [19]. Following a new CVD event, patients with RA have a 17.6% 30-day CVD mortality risk compared to 10.8% in the non-RA population [20]. These patients had an odds ratio (OR) and 95% confidence interval (CI) of 1.6, 1.2-2.2 for increased CVD mortality after 30-days of an myocardial infarction (MI) compared to the non-RA population [20].

Similar findings were observed in a meta-analysis of 111,758 patients with 22,927 cardiovascular events that found a 50% increased risk of CVD death among patients with RA compared to the general population [21]. Another meta-analysis reported a 60% increase in CVD death compared to non-RA subjects [22]. Results from Nurses’ health study found that women with RA had 45% increased CVD mortality with a hazard ratio (HR) of 1.5, 95% CI 1.1-1.8, compared to non-RA women [16]. Though the relative risk (RR) and rates of CV mortality may vary among different data sources owing to differences in patient population, duration of follow up, measurement of outcome and missing data on specific cause of death, these studies still considerably support the increase CVD mortality that exists among patients with RA [23].

CVD mortality has been associated with level of inflammation, HLA–DRB1*0404 [10], use of glucocorticoids [24] and presence of RA autoantibodies [25, 26], and can possibly be reduced by effective RA treatments [27, 28]. The time trend studies of overall mortality and CVD specific mortality in RA showed persistently increased CVD mortality except for some recent data suggesting a downward trend. A 2007 study by Gonzalez et al. demonstrated a widening gap between overall mortality in RA compared to general population [29]. A recent (2014) analysis from United Kingdom (U.K.) based cohort, Norfolk Arthritis Register, included 2517 patients with early inflammatory arthritis with 16,485 person-years of follow-up. In this study, CVD mortality decreased with time in the first seven years from recruitment in this register, but was increased among patients who were antibody-positive [25].

In a population-based incident RA cohort from Canada, Lacaille et al. reported improvement in overall mortality and a similar 5-year CV mortality in RA patients with disease onset in 2001-2006 to non-RA patients [30]. Another study showed improved CVD mortality in an RA cohort from 2000 to 07 (2.7%, 95% CI 0.6–4.9%) compared to patients diagnosed in 1990–99 (7.1%, 95% CI 3.9–10.1%) suggesting a decline in CVD mortality in more recent years [31]. It must be noted that results of this particular study were based on only 315 RA patients from a single county in the United States of America (U.S.A) with 8 deaths from CVD, which could be a result of regional differences and may not represent the actual CVD mortality among patients with RA at a population level [31].

Many of the studies that showed a decrease in CVD mortality in the U.S. were not population-based. In order to confirm an actual decrease in CVD mortality, larger population-based studies with longer follow up are needed. Overall, the data thus far remains robust in support of a current and persistent increased CVD mortality among patients with RA [25, 32–36].

### Traditional cardiovascular risk factors

#### Hypertension (HTN)

Hypertension (HTN) is a well-established risk factor for developing cardiovascular disease [37] with a prevalence of 29% in the general population [38]. Prior studies report a wide range of prevalence of hypertension in patients with RA ranging between 3.8%-73% [39–44]. Similar to the general population, hypertension is detrimental for CVD risk among patients with RA and is an independent predictor of CVD events [41, 45]. A meta-analysis of longitudinal studies found an 84% increased risk of myocardial infarction among patients with RA with hypertension compared to non-hypertensive patients with RA (RR 1.8, 95% CI 1.4-2.5) [46].

Multiple factors may impact blood pressure in patients with RA including inflammation, physical inactivity, and drugs [40]. Increased arterial stiffness and reduced elasticity of blood vessels is seen in patients with RA [47, 48]. Studies in animal models suggest an association between ongoing inflammation and hypertension [49]. Although, the exact underlying mechanisms remain to be fully understood. This association can be seen clinically in thedata from the Women’s Health Study, an ongoing randomized, double-blind, placebo-controlled trial of low-dose aspirin and vitamin E for the primary prevention of CVD and cancer, that evaluated women with incident hypertension. This study shows that high C-reactive protein (CRP), is associated with increased risk of developing hypertension among healthy women [50, 51]. Finally, medications that are often prescribed to patients with RA, such as non-steroidal anti-inflammatory drugs (NSAIDs) and glucocorticoids, are associated with increased risk for HTN [52, 53].

Despite high prevalence of HTN and associated cardiovascular risk, HTN in rheumatoid arthritis is under-recognized and suboptimally treated [54–56]. Results reported from a U.K. based study showed that among 221 patients with RA and elevated CVD risk, 32% had a systolic blood pressure > 140 mmHg and only 23% were treated with antihypertensive drugs [55]. Among these patients with RA treated with antihypertensive drugs, 50% still had a systolic blood pressure > 140 mmHg [55]. Furthermore, there is a gap in coordinated care for the management of hypertension for patients with RA between rheumatologists and primary care physicians. While rheumatologists routinely screened for hypertension, only 31% of them initiated HTN treatment for these patients [57].

A study from a large academic center used electronic health records to identify patients with hypertension. They identified 14,974 patients with undiagnosed hypertension who were seen regularly in the primary care setting [56]. Among these, 201 patients had RA. When compared to non-RA controls, RA patients had 29% lower hazard of receiving a diagnosis of hypertension at mean follow up of 14 months (HR 0.7, 95%CI 0.6–0.9) even though their number of visits to a primary care physician was equivalent to that of patients without RA [56]. This has significant clinical implications as uncontrolled HTN may lead to a higher number of CVD events. In a study by Singh et al. investigators used cardiovascular risk prediction models from the Framingham Heart Study. This study showed that a 20 mmHg increase in systolic blood pressure in RA patients was associated with 1572 additional ischemic heart disease events yearly [58]. Given the heightened CVD risk imparted by HTN in RA patients, this comorbidity needs more attention for appropriate screening and optimal treatment.

#### Insulin resistance/metabolic syndrome

Metabolic syndrome has been defined in the general population as having three of five elements including obesity, elevated triglycerides, low high-density lipoproteins (HDL) cholesterol, high systolic and diastolic blood pressure, and elevated fasting glucose [59]. Metabolic syndrome increases CVD risk by 2 fold in general population [60]. Da Cunha et al., in a study conducted in Brazil, found a larger number of patients with RA with metabolic syndrome when compared to healthy non-RA controls (39% vs 19%) [61]. The authors also noted increased prevalence of waist circumference, hypertension, and increased fasting glucose in patients with RA when compared with controls [61].

A recent meta-analysis of prevalence studies of metabolic syndrome in RA patients showed a prevalence of 30.7% (95% CI 27.9-33.4) [62]. Insulin resistance is a key factor for the development of CVD risk in metabolic syndrome [63]. Glucocorticoids (GC), commonly used to treat RA related symptoms, promote insulin resistance; each 5 mg increase of current oral GCs is associated with a 25–30% increased risk of type 2 diabetes mellitus (DM) [64]. Insulin resistance and type 2 DM are linked with elevation of inflammatory markers such as erythrocyte sedimentation rate (ESR), CRP, and RA disease activity [65, 66]. The elevation of these inflammatory markers, in addition to the inflammation caused by RA, further increases the risk for developing atherosclerosis.

#### Body weight/obesity

Body mass index (BMI), derived from mass and height of an individual (kg/m^2^), is a commonly used measure for body composition in RA and non-RA individuals alike. Obese individuals (> 30 kg/m^2^) [67] have a two to three times higher mortality than normal weight individuals [68]. Obesity is independently associated with CVD burden as well as other CVD risk factors such as hypertension, dyslipidemia, insulin resistance etc. [69]. It is also associated with endothelial dysfunction and promotion of atherosclerosis [70]. Similar to the general population, obesity contributes to cardiovascular morbidity in patients with RA [71]. In patients with RA, it is independently associated with other CVD risk factors [72]. and also predicts 10-year CVD risk [71, 72]. Adipose tissue is a source of inflammatory factors including interleukin-6, tumor necrosis factor-alpha, and CRP which induce a state of low-grade inflammation that contributes to CVD risk [73].

Paradoxically, a low BMI in RA (< 18.5 kg/m^2^) has been associated with high CVD risk in these patients [74]. A possible explanation for this phenomenon of low BMI is rheumatoid cachexia [75]. A chronic inflammatory state such as the one that occurs in RA can cause alterations in body composition. Individuals with RA may lose lean muscle mass and accumulate excess fat. This makes it challenging to use BMI as a marker of body composition because it cannot distinguish the proportion of adipose tissue and muscle. It remains unclear how to best identify those patients with RA who have a disproportionate adipose tissue to muscle ratio. A past study found that, for a given body fat content, patients with RA had a significantly lower BMI by almost 2 kg/mg^2^ compared to general population. Investigators of this study proposed that the BMI cut off for RA patients should be reduced to 23 kg/m^2^ for overweight and 28 kg/m^2^ for obesity respectively [66]. While it is an interesting observation, these cut off points have not been used extensively in population-based cohorts to determine whether these are indeed predictive of CVD events in patients with RA. Alternative measures that have been proposed include waist circumference and waist to hip ratio but thus far they have not been proved superior to BMI in assessing obesity-related comorbidity [76]. Further research is needed to identify the optimal way to define obesity in patients with RA.

#### Smoking

Patients with RA who smoke have aggressive disease and worse clinical outcomes [77]. Despite the associated hazards, a meta-analysis determined that the prevalence of smoking was higher in patients with RA compared with controls (OR 1.6, 95%CI 1.4-1.8) [78]. In the general population, cigarette smoking is associated with CVD [79]. Although among patients with RA its impact on CVD is less clear, some studies in the past showed that there was a weak association between smoking and CVD in patients with RA [5, 80], However, it is possible that this weak association is attributed to under–reporting of smoking status [81] or index event bias (a type of selection bias that occurs when multiple risk factors contribute to the risk of the index outcome (disease) as well as disease sequela) [82].

It is known that cigarette smoking is associated with rheumatoid factor positivity [83], production of anti-citrullinated antibodies (CCP) [84], increased disease severity [77], and poor response to treatment [85], all of which have been associated with CVD morbidity in patients with RA [25, 26, 86, 87]. More recent data have shown that smoking is associated with CVD risk. In a large longitudinal study from the Veterans Health Administration (VHA), (37,568 patients with RA and 896 incident hospitalized myocardial infarction) “current smoking” was associated with an increased risk of myocardial infarction by 42% vs. “never smoker” (HR 1.4, 95% CI 1.1-1.8) [88]. Another study of 5638 patients with RA with no prior CVD who were followed for 5.8 years found that smoking had the highest population attributable risk (PAR) for CVD across different CVD risk factors including RA disease activity (PAR for smoking = 23.7%) [89]. Moreover, a recent meta-analysis of longitudinal studies noted a 50% increased risk of CVD events in smokers compared to non-smoker RA patients (*n* = 2056, RR 1.5, 95% CI 1.3-1.8) [46]. A significant number of patients with RA continue to smoke therefore, interventions for smoking cessation should be applied not only to improve RA disease activity but also to ameliorate their overall CVD risk.

#### Lipids

In the general population, the atherogenic lipid profile is considered to be high total cholesterol (TC), low-density lipoprotein cholesterol (LDL-C) and low high-density lipoprotein cholesterol (HDL-C). Dyslipidemia is commonly seen in patients with RA and is linked to increased cardiovascular disease [90]. A retrospective study of 1078 patients showed that lipid changes (higher TC, lower HDL-C, higher triglycerides) may be present even before the onset of RA [91]. High levels of lipoprotein (a), which is structurally similar to LDL-C and is atherogenic in nature, have also been reported in patients with RA [92, 93].

The relationship of lipids in patients with RA is more complex than in non-RA individuals because of the interplay of cholesterol with inflammation. Cholesterol levels decrease in the presence of active inflammation. The Third National Health and Nutrition Examination Survey (NHANES) compared lipid profiles of 128 patients with RA aged 60 and older to non-RA controls and found that patients with RA who were not on DMARDs or glucocorticoids had significantly low levels of HDL cholesterol [94]. Similarly, low TC and LDL-C levels were seen in patients with active RA while the rate of having a myocardial infarction remained 1.6 times higher than patients without RA [95, 96]. This has been defined as the RA ‘lipid paradox’ [95]. High CRP among patients with RA representing high level of inflammation correlates with lower TC, LDL-C and HDL-C while at the same time that high CRP is associated with increased CVD risk [97, 98]. While the exact mechanism for the lipid paradox remains unknown, genetic factors, reduced lipid synthesis, increased clearance as well as cholesterol consumption as an essential substrate to develop an inflammatory response have been implicated as causes for the low cholesterol levels [8, 99, 100]. It has also been observed that RA therapies increase the lipid levels while reducing inflammation (See Table 1) [101]. These changes gathered special attention during the clinical trials of tocilizumab (TCZ), an interleukin (IL)-6 receptor blocker. A significant increase in lipid levels was observed in patients who received TCZ [102, 103]. There are ongoing studies to determine whether these changes are detrimental for CVD risk and if so, to what extent. A similar pattern of lipid changes was also seen with other RA therapies such as DMARDs, and tumor necrosis factor (TNF) alpha inhibitors (see Table 1) which suggests that these changes are not only a result of an intrinsic mechanism of action (IL-6 blockade) but also from decreased inflammation.

**Table 1:** Summary of Changes in Lipid Profiles with Rheumatoid Arthritis Therapies

Study or Author	RA drug	Change in TC (mg/dL)	Change in LDL-C (mg/dL)	Change in HDL-C (mg/dL)	Notes
Yamanaka et al. [171]	TCZ	↑ 12.9	n/a	n/a	Concurrent DMARD use
Yazici et al. [172]	TCZ	↑ 25.9	↑ 17.8	↑ 3.5	Concurrent DMARD use
Genovese et al. [103]	TCZ	↑ 30.9	↑ 3.8	↑ 19.3	Concurrent DMARD use
Gabay et al. [161]	TCZ	↑30.5	↑20.1	↑5.4	Change from baseline to week 8
	Adalimumab	↑6.6	↑2.7	↑2.7	
Tam et al. [173]	Infliximab	↑23.2	↑12.4	↑5.8	Change from baseline to week 14
Kirkham et al. [174]	Golimumab	↑8.0	↑8.0	↑3.0	
Morris et al. [175]	Hydroxychloroquine	↓ 7.7	↓ 7.6	↑ 1.0	
Navarro-Millán et al. [176]	Methotrexate with Etanercept	↑56.8	↑31.4	↑19.3	After 6 months of treatment
	Triple therapy^a^	↑53.0	↑28.7	↑22.3	
	Methotrexate	↑57.3	↑30.0	↑20.6	
Charles-Schoeman et al. [177]^b^		↑ week 24	↑ week 24	↑ week 24	Trend of lipids with treatment
		↓week 102	↓week 102	↓week 102	
Novikova et al. [178]	Rituximab	↑19.7	↑4.6	↑12.0	After 6 months of treatment

*TC* total cholesterol, *LDL-C* low-density lipoprotein cholesterol, *HDL-C* high-density lipoprotein cholesterol, *TCZ* tocilizumab

^a^Triple therapy=methotrexate plus sulfasalazine plus hydroxychloroquine, DMARD = disease modifying anti-rheumatic drugs

^b^Absolute numerical changes not available

Besides the quantitative changes in lipids, inflammation also impacts the qualitative aspect of the cholesterol. The level of inflammation may determine how much impact LDL-C has on CVD risk. For example, LDL-C had more impact on the CVD risk when ESR was more than 30 mm/h [95]. Furthermore, inflammation also affects anti-oxidant capacity of HDL-C. HDL-C under normal circumstances is responsible for inhibiting oxidation of LDL-C and efflux of cholesterol from vessel walls [104]. In a state of inflammation, HDL-C gets altered, losing its ability to remove cholesterol from atherosclerosis, and indeed becoming pro-atherogenic [105]. HDL-C is also reduced in patients with RA, resulting in a high atherogenic index of total­cholesterol:HDL­C ratio [101, 106]. RA treatment, improves HDL-C function as a consequence of decreasing inflammation, which highlights the importance of controlling RA disease activity to improve lipid profiles and decrease overall CVD risk [107].

#### Physical inactivity and cardiopulmonary fitness

Physical inactivity is associated with higher risk of myocardial infarction in the general population according to the INTERHEART case–control study [108]. Data from 33 large prospective cohorts demonstrated a 35% relative risk reduction in CVD related death associated with being physically active [109]. Unfortunately, several studies indicate that patients with RA are frequently inactive [110–112]. This is partly due to pain and fatigue [113], lack of motivation [114], and lack of patient understanding of the negative impact of physical inactivity [115].

A recent meta-analysis showed that CVD morbidity was not increased with physical inactivity among RA patients (RR 1, 95%CI 0.7-1.3) [46]. However, the results must be interpreted with caution because this meta-analysis included only two studies, both of which had cross sectional designs. A cross sectional study examined the impact of physical activity on CVD risk profile in RA patients. Levels of physical activity were assessed in 65 patients using a questionnaire. After adjusting for age, weight, sex, smoking status, and RA disease activity, physically active patients with RA had significantly lower systolic blood pressure, cholesterol levels, low density lipoprotein, homocysteine, Apolipoprotein B, von Willebrand Factor, and Type-I plasminogen activator inhibitor antigen [116]. This suggests that CVD risk profile of patients with RA can be improved by implementing increased physical activity. Data from a systematic review of randomized clinical trials of exercise programs among patients with RA showed that exercise improved aerobic and muscle strength among these patients [117]. The benefit on decreasing CVD risk still requires more direct and specific evaluation since none of these trials evaluated this relationship [117].

There is accumulating clinical data that shows improved CVD risk parameters with exercise in RA. Forty patients with RA were divided into an exercise group who received 6 months of tailored aerobic and resistance exercise and a control group who received only information of exercise benefits. Significant improvement in the endothelial function parameters was noted in the exercise group compared to the control group. This suggests that exercise may reduce CVD risk by impacting endothelial dysfunction, though long-term effect of exercise intervention on this parameter needs further evaluation [118]. Other studies show that exercise can reduce CRP levels [119] and also has an anti-atherogenic effect, which further elaborates the impact of exercise on CVD risk [119, 120].

Low levels of cardiopulmonary fitness, measured by the maximal oxygen uptake (VO_2_max) test is associated with CVD and all-cause mortality [121–123]. It has been reported that patients with RA have low cardiopulmonary fitness [121]. A recent cross-sectional study evaluated the association of VO_2_max with CVD risk in the RA population [124]. Results showed that patients with RA not only had lower VO_2_max levels, but also that those with higher levels of VO_2_max had better cardiovascular risk profiles. There is evidence that cardiopulmonary fitness in RA can be improved with aerobic and resistance exercise intervention; thus, providing an exercise program to patients with RA is a useful tool to attenuate CVD risk [125]. Based on current evidence, RA patients should be encouraged to exercise not only to improve physical function but also to reduce cardiovascular disease.

### RA related factors

#### Inflammation

Atherosclerosis is no longer thought to be a simple process of lipid accumulation in blood vessels. There is evidence that systemic inflammation plays a pathogenic role in the development of accelerated atherosclerosis. A study found that even in healthy men, inflammation measured by elevated inflammatory markers was associated with increased CVD risk [126]. Atherosclerotic plaque formation begins with endothelial dysfunction, after which pro-inflammatory cytokines and adhesion molecules are released. Inflammatory cells then enter the blood vessel wall along with LDL molecules because of increased endothelial permeability. LDL is oxidized and taken up by the macrophages, which later become foam cells. This is followed by smooth cell proliferation and neovascularization which ultimately cause the thickening of the blood vessel and plaque formation [12].

Past studies have shown that endothelial dysfunction is impaired in RA patients [127] with a magnitude equivalent to that of diabetes, an independent CVD risk factor [18]. Circulating inflammatory substances and autoantibodies, such as anti-CCP and rheumatoid factor, are associated with endothelial dysfunction [128, 129]. A recent systematic review of randomized clinical trials suggested that endothelial dysfunction in RA can be improved with TNF alpha-blockers, but the conclusion was based on small observational studies and further randomized controlled data is needed to validate these findings [130]. Similarly, inflammatory cytokines such as IL-6, IL-18, and TNF-alpha, which are typically elevated in rheumatoid arthritis, have been associated with cardiovascular disease [131]. Markers of inflammation in patients with RA such as ESR and CRP are associated with intimal media thickness, a surrogate for atherosclerotic disease [132–134]. There is also development of pro-atherogenic HDL in the setting of inflammation from RA [107, 135]. Inflammation thus significantly contributes to CVD risk in patients with RA in addition to traditional CVD risk factors.

#### NSAIDs and glucocorticoids(GCs)

A broad use of NSAIDs and GCs is common among patients with RA by virtue of their anti-inflammatory properties. However, these drugs have implications pertaining to CVD risk.

GCs are associated with insulin resistance [65], hypertension [53], obesity, hyperlipidemia [136] and DM [64], all of which are associated with development of CVD. They are associated with CVD mortality in a dose dependant fashion [24]. On the contrary, there are studies that suggested that GCs may prove beneficial in reducing CVD risk by controlling inflammation [42]. Robust randomized trials to prove this notion are lacking and EULAR currently recommends keeping GCs at a minimum dosage.

NSAIDs have been associated with CVD risk in the general population, but whether they augment CVD risk in RA needs to be well established. A systematic review and meta-analysis showed that NSAIDs increase risk of CVD events in RA [137]. However, the effect was mainly driven by rofecoxib and not from non-selective NSAIDs or celecoxib, another cyclooxygenase 2 inhibitor. Rofecoxib has now been withdrawn from the market and the recent PRECISION trial found similar CVD safety of celecoxib to ibuprofen and naproxen in patients with arthritis (~ 10% of total population had RA) [138]. In the Danish cohort, investigators found a significantly lower CVD risk associated with NSAIDs in RA compared to non-RA [139]. The evidence as of yet is not strong enough to contraindicate the use of NSAIDs in patients with RA and the recommendation is to use them cautiously in this population [13]. A meta-analysis found naproxen to be least harmful for CVD safety [140]. Nevertheless, further research is needed to understand the impact of NSAIDs in RA patients, particularly in patients with pre-existing CVD risk factors.

### Cardiovascular risk assessment

Cardiovascular risk assessment is intended to identify patients who are at high risk of developing CVD in the future so that preventive strategies could be implemented proactively. Several algorithms that quantify this risk are available to use in the general population, which are also applicable to patients with RA. These models utilize traditional parameters such as age, sex, blood pressure, smoking status, cholesterol levels and presence of diabetes mellitus to compute a risk for CVD in these patients [141]. There are some noteworthy challenges to the use of these algorithms for patients with RA. These models do not account for the increased CVD risk associated with RA inflammation. For instance, the Framingham Score and even the 10-year Pooled Cohort Risk Equation do not take into consideration the effect that RA has on CVD risk as these models do for DM [141, 142]. This is despite the fact that both diseases are independent risk factors for CVD [17]. Therefore it appears that, these instruments can underestimate CVD risk in patients with RA, which has led to multiple studies to determine how more accurataly RA-specific instruments compared to those based on the general population, can predict CVD risk in these patients. Since inflammation and RA disease activity fluctuate over time, the development of a precise CVD prediction model is even more challenging. These changes suggest that CVD risk in patients with RA is more dynamic rather than fixed. Further studies are needed to determine the importance of the changes in RA disease activity and its impact on calculating CVD risk. Nevertheless, using current CVD risk prediction models still provides a valuable starting point to initiate cardiovascular disease primary risk prevention.

Several algorithms are available to stratify CVD risk in a patient. The SCORE (Systematic Coronary Risk Evaluation) CVD death risk score was developed from 12 European cohort studies and is used in European countries [143]. It calculates 10-year risk of any first fatal atherosclerotic event. In the United States, the American College of Cardiology/American Heart Association (ACC/AHA) guildeines on the tratment of blood cholesterol recommends initiation of a lipid-lowering agent and lifestyle modifications if the 10-year CVD risk is => 7.5 [144]. The Reynolds risk score was developed from prospective cohorts of men and women without diabetes [145, 146]. It does account high sensitivity CRP into the equation, so theoretically it can better predict CVD risk in RA. However, CRP is more sensitive for short-term changes in inflammation. A clinical study found that, despite accounting for CRP, the Reynolds risk score substantially underestimated CVD risk in patients with RA (both men and women) [147]. The QRISK-2 calculator is the only calculator that takes RA into account as a CVD risk factor in addition to traditional risk factors [148]. However, studies have shown that QRISK2 may overestimate the CVD risk in patients with RA [149, 150].

Recently, a new cardiovascular risk calculator, called the Expanded Cardiovascular Risk Prediction Score for Rheumatoid Arthritis (ERS-RA) was developed for RA patients using a cohort for 23,605 patients with RA from the Consortium of Rheumatology Researchers of North America (CORRONA) [14, 15]. It includes RA-related variables such as Clinical Disease Activity Index (CDAI) > 10 versus ≤10), disability (modified Health Assessment Questionnaire disability index > 0.5 versus ≤0.5), daily prednisone use and disease duration (≥10 versus < 10 years) in addition to traditional CV risk factors (i.e., age, sex, diabetes mellitus, hypertension, hyperlipidemia, and tobacco use). In this model, actual blood pressure and cholesterol values were not available. Investigators then accounted for these traditional risk factors based on physician reported diagnosis for HTN and hyperlipidemia or the use of medications for either of these conditions. External validation is still needed for this calculator to know whether it could be applied to the general U.S. and non-US populations [14].

A recent study combined data from seven RA cohorts from the U.K., Norway, Netherlands, the United States of America (U.S.A), South Africa, Canada and Mexico and compared the performance of QRISK2, the EULAR multiplier and ERS-RA to risk calculators for the general population: ACC/AHA, Framingham Adult Treatment Panel III, Framingham risk score-Adult Treatment Panel (FRS-ATP) and the Reynolds Risk Score [15]. The study found that RA risk calculators did not perform better than general population risk scores [15]. Hence, it is reasonable to apply these prediction models the same way as they are applied in the general population while specific prediction models for RA are being developed and validated. European League Against Rheumatism (EULAR) 2016 CVD treatment guidelines for RA recommend applying a multiplication factor of 1.5 to the scores that does not account for RA by default [13]. The guidelines also recommend performing CVD risk screening once every 5 years and treating modifiable CVD risk factors in order to decrease the risk. EULAR recommendations is to use national guidelines applicable to the general population to determine which CVD risk prediction model be used. However, if national guidelines are not available, the SCORE model can be used for CVD risk assessment, at least according to the European guidelines.

### Management

#### RA disease activity and the role of RA therapy

Studies have shown that lowering disease activity also decreases CVD events. A 10-point decrease in the clinical disease activity index (CDAI) was associated with a 21% reduction in CVD risk (95% CI 13.0-29.0) [86]. Another study showed that low disease activity measured by the Disease Activity Score-28 joint count DAS28 (≤3.2) was associated with reduced CVD risk compared to high disease activity (DAS > 3.2) [151]. Recent data from the Brigham and Women’s Hospital Rheumatoid Arthritis Sequential Study (BRASS), a prospective observational RA cohort, highlights the improvements in HDL-C efflux capacity with reductions in high sensitivity CRP [152].

Multiple studies have shown that the management of CVD risk should rely on tight RA disease control regardless of type of therapy used. Ljung et al. showed that RA patients on TNF inhibitor therapy who had good EULAR response had 50% lower risk of acute coronary syndrome compared with non-responders [87]. However, EULAR moderate responders had equal risk to that of EULAR non-responders, implying that optimal disease control is needed to reduce CVD risk not just disease control or being on a TNF inhibitor. The number of patients with RA that achieve remission or low disease activity remains low with a prevalence of remission fluctuating between 8 and 20% [153–155]. Given that only a small number of patients achieve clinical remission, it is also important to target traditional modifiable CVD risk factors to ameliorate CVD risk in these patients.

Use of anti-rheumatic therapy is associated with reduced CVD risk. A large meta-analysis of 10 cohort studies showed an 18% to 21% decrease in the risk for CVD related events (myocardial infarction, coronary heart disease, sudden death, and/or stroke) with use of methotrexate (MTX) [156]. MTX may improve HDL-C’s anti-inflammatory function [157]. There is an ongoing clinical trial that is evaluating the effect of methotrexate on cardiovascular outcomes in a high CVD risk population without RA [158].

In terms of CVD outcomes, a systematic review and meta-analysis of observational cohorts and randomized controlled trials (RCTs) reporting on cardiovascular events in RA patients showed a decrease in CVD risk with the use of anti-TNF therapy [159], but the results of the meta-analysis were not statistically significant. Del Rincón et al. demonstrated that, even in the presence of a high level of inflammation (represented by ESR), anti-TNF therapy and MTX decreased the progression of intima-media thickness (IMT) [132]. The main limitation of the study was the lack of a non-RA control group.

Interleukin 6 (IL-6) blocker tocilizumab is of particular interest with respect to CVD risk because of their potentially adverse effects on the lipid profile. However, data from a phase 4 clinical trial comparing cardiovascular safety of tocilizumab vs etanercept in patients with RA showed that the rate of major CVD events with tocilizumab was low and comparable to that of etanercept (83 tocilizumab arm versus 78 in etanercept arm, (HR 1.1; 95% CI 0.8, 1.4) [160]. A post hoc analysis from a clinical trial of RA patients that received intravenous tocilizumab or adalimumab noted that LDL-C and HDL-C increased with both treatments but the magnitude of these changes was higher in the tocilizumab group. While this data suggest that the impact of different therapies on lipid profiles is not equivalent, the observation across studies is that RA treatments increase lipid levels [161]. Further studies are needed to understand the implications of these changes on cardiovascular risk in RA patients, but the data reported to date do not suggest that these changes are detrimental toward CVD risk.

#### Traditional risk factors:

Several studies showed that primary lipid screening was performed in less than half of patients with RA [162, 163]. It is often questioned which physician (rheumatologist, primary care) should take ownership of performing CVD risk management. In the most recent guidelines, EULAR strongly encouraged rheumatologists to take ownership of the management of this risk factor. National guidelines for the general population should be used to manage traditional risk factors such as hypertension, diabetes and hypercholesterolemia. Lipid management should be carried out similar to the general population. Given that active inflammation in RA can alter the lipid levels, lipid testing should be carried out when a patient’s disease activity is stable or in remission [13].

Drugs such as non-steroidal anti-inflammatory drugs and glucocorticoids exert deleterious effects on blood pressure, lipid profile and glucose tolerance and therefore should be kept to minimum [24, 164]. Lifestyle changes should be recommended to all patients with emphasis on a diet without trans fatty acids and high content of fruits and vegetables, regular exercise and smoking cessation. A structured exercise program should be offered as it improves cardiorespiratory fitness and reduces CVD risk [125].

Management of hypertension should be carried out as in the general population. There is no evidence that the treatment thresholds should differ from the general population [37]. Current guidelines for prevention and management of hypertension in adults recommend anti-hypertensive medication for primary prevention in adults with estimated 10-year atherosclerotic cardiovascular disease => 10% and an average systolic blood pressure => 130 mmHg or diastolic blood pressure => 80mm Hg [165]. Lipid management should be carried out similar to the general population.

Statins are effective at improving lipid profiles [166–168]. Similar to general population, statins reduce CVD risk in RA as well [169]. A multicenter, double blind prospective study of 2986 patients with RA found a 34% reduction in cardiovascular events after treatment with atorvastatin compared to placebo. The results were not statistically significant because the trial was abandoned early because of a lower than anticipated event rate [170]. A recent study examined the impact of lowering LDL-C in two cohorts of RA patients (*n *= 1522 and 1746 respectively) who were matched with a control group comprised of general population and patients with osteoarthritis. All these patients had a hyperlipidemia diagnosis and a statin prescription. It was noted that lower LDL-C levels were associated with reduction of cardiovascular events [169]. Regardless of the “lipid paradox” in RA (low lipid levels but higher incidence of CVD) and the changes in lipid profiles observed with RA treatment, statins should be used in accordance with CVD treatment guidelines for primary prevention in this population. Still, this approach is not regularly used in clinical practice, possibly because of “normal” or “abnormally low” lipid profiles in patients with RA in the presence of high disease activity and the lack of recognition of CVD risk imparted by RA [163]. The practice can be enhanced by a more unanimous agreement of when and how statins should be initiated in patients with RA.

According to the ACC/AHA cholesterol treatment guidelines, statins should be initiated for primary prevention if the calculated 10-year CVD risk ≥7.5% for patients between 40 and 75 years of age in the U.S.A [144]. Once a CVD event has occurred (secondary prevention), every patient with RA should be initiated on a statin. In other countries (such as European countries), statin initiation can be carried out per national guidelines of CVD management for the general population [13].

## Conclusion

Cardiovascular burden is significantly increased in rheumatoid arthritis. In addition to RA disease activity control, management of traditional risk factors for CVD is imperative. A multidisciplinary approach should be sought where primary care practitioners, rheumatologists and cardiologists can work together to improve cardiovascular outcomes and reduce mortality among patients with RA.

## Abbreviations

ACC/AHA: American College of Cardiology/American Heart Association
BMI: Body mass index
BRASS: Brigham and Women’s Hospital Rheumatoid Arthritis Sequential Study
CCP: Cyclic citrullinated peptide
CDAI: Clinical disease activity index
CI: Confidence interval
CORRONA: Consortium of Rheumatology Researchers of North America
CRP: C reactive protein
CVD: Cardiovascular disease
DAS28: Disease Activity Score-28 joint count
DM: Diabetes Mellitus
DMARD: Disease modifying anti-rheumatic drugs
ERS-RA: Expanded Cardiovascular Risk Prediction Score for Rheumatoid Arthritis
ESR: Erythrocyte Sedimentation rate
EULAR: European League Against Rheumatism
FRS-ATP: Framingham risk Score-Adult Treatment Panel
GC: Glucocorticoid
HDL: High density lipoprotein
HDL-C: High density lipoprotein cholesterol
HR: Hazard ratio
HTN: Hypertension
IL: Interleukin
IMT: intima-media thickness
LDL-C: low-density lipoprotein cholesterol
MI: Myocardial infarction
MTX: Methotrexate
NHANES: National Health and Nutrition Examination Survey
NSAIDs: non-steroidal anti-inflammatory drugs
OR: Odds Ratio
PAR: Population attributable risk
RA: Rheumatoid arthritis
RR: Relative risk
SCORE: Systematic Coronary Risk Evaluation
TC: Total cholesterol
TCZ: Tocilizumab
TNF: Tumor necrosis factor
U.S.A: United States of America
VHA: Veterans Health Administration
VO_2_max: maximal oxygen uptake
